# An Unknown Non-denitrifier Bacterium Isolated from Soil Actively Reduces Nitrous Oxide under High pH Conditions

**DOI:** 10.1264/jsme2.ME20100

**Published:** 2020-12-05

**Authors:** Yuta Takatsu, Toshizumi Miyamoto, Yasuyuki Hashidoko

**Affiliations:** 1 Research Faculty of Agriculture, Hokkaido University, Kita 9, Nishi 9, Kita-ku, Sapporo, Hokkaido 060–8589, Japan

**Keywords:** bacteria, farmland soil, N_2_O, N_2_O reduction, *nosZ*

## Abstract

A nitrous oxide (N_2_O)-consuming bacterium isolated from farmland soil actively consumed N_2_O under high pH conditions. An acetylene inhibition assay did not show the denitrification of N_2_ to N_2_O by this bacterium. When N_2_O was injected as the only nitrogen source, this bacterium did not assimilate N_2_O. A polymerase chain reaction demonstrated that this bacterium did not have the typical *nosZ* gene. This bacterium belonged to *Chitinophagaceae*, but did not belong to known families that include bacteria with the atypical *nosZ*. This is the first study to show that a non-denitrifier actively reduces N_2_O, even under high pH conditions.

Although carbon dioxide is a well-known greenhouse gas (GHG), other GHG also influence climate change (Montzka *et al.*, 2011). Among these GHG, nitrous oxide (N_2_O) has a major impact on global warming. N_2_O absorbs infrared radiation, and its potential to cause global warming is 298-fold that of carbon dioxide and, thus, is regarded as the most important ozone-depleting substance in this century (Ravishankara *et al.*, 2009; Montzka *et al.*, 2011). The emission of N_2_O from agricultural soil is accelerated by the addition of large amounts of nitrogen-containing fertilizers to farmlands, and accounts for 60% of the atmosphere (Mosier *et al.*, 1998; Zhou *et al.*, 2015). Various methods have been attempted to mitigate N_2_O emissions. Recent studies reported that N_2_O emissions may be suppressed by the addition of a substance used as an agrochemical (Obia *et al.*, 2015; Abbruzzini *et al.*, 2019; Takatsu *et al.*, 2019). However, the use of agrochemicals is associated with a number of issues, such as the loss of soil biodiversity and the persistence of soil chemicals (Stolte *et al.*, 2016; Silva *et al.*, 2018), thereby necessitating other methods. Therefore, N_2_O-reducing microorganisms have been attracting increasing attention (Hallin *et al.*, 2018).

N_2_O is emitted from soil into the atmosphere through the processes of nitrification and denitrification by soil microorganisms, which are major sources of N_2_O in soil (Skiba and Rees, 2014). In the denitrification pathway, complete denitrifiers (NO_3_^–^/NO_2_^–^ → NO → N_2_O → N_2_) and incomplete denitrifiers (NO_3_^–^/NO_2_^–^ → NO → N_2_O) contribute to the emission of N_2_O. Complete denitrifiers possess the *nosZ* gene, which encodes N_2_O reductase (Zumft, 1997; Wunsch and Zumft, 2005). Furthermore, some non-denitrifying N_2_O-reducing microorganisms lack the pathway for the conversion of NO_3_ to N_2_O, but have the capacity to convert N_2_O to N_2_ (Payne *et al.*, 1982; Simon *et al.*, 2004). Therefore, non-denitrifying N_2_O-reducing microorganisms have the potential to be true N_2_O sinks without contributing to N_2_O production (Hallin *et al.*, 2018). NosZ protein phylogeny has two distinct groups, clade I and II *nosZ* (Hallin *et al.*, 2018). These clades have been reported as typical and atypical *nosZ* (Sanford *et al.*, 2012). Clade I *nosZ* comprises alpha-, beta-, or gamma-proteobacteria, while clade II *nosZ* consists of a large range of archaeal and bacterial phyla (Jones *et al.*, 2013). Non-denitrifying N_2_O-reducing microorganisms belong to clade II and possess abundant diversity in all ecosystems (Hallin *et al.*, 2018).

Complete denitrifiers utilize the N_2_O present in soil gas as the final electron acceptor in the nitrate respiratory system and emit N_2_ as the final product into the atmosphere (Hutchins, 1991; Wunsch and Zumft, 2005). N_2_O is used to promote cell survival, even in the absence of oxygen (Park *et al.*, 2017). Based on the assimilation of N as a nutrient, when N_2_O is abundant, from the perspective of activation energy, it is more efficient in the assimilation of N_2_O than N_2_ fixation (Kryachko *et al.*, 2001). Available N (NO_3_^–^ and NH_4_^+^) in soil is limited, even in relatively fertile soils because these nitrogen sources are competitively assimilated by plants and other microorganisms (Kaye and Hart, 1997). In terms of a survival strategy for bacteria, the assimilation of N_2_O is advantageous when N_2_O is abundant. Therefore, some bacteria that positively absorb N_2_O for assimilation may exist; however, this has not yet been demonstrated.

Therefore, the purpose of the present study was to search for a bacterium in soil that consumes N_2_O. We hypothesized that some bacteria among N_2_O-consuming microorganisms in farmland soil may assimilate N_2_O when it is abundant through the denitrification process. By detecting changes in N_2_O concentrations in gas chromatography vials injected with N_2_O before incubations, strains with the potential to consume N_2_O were screened among bacteria isolated from farmland soil. Furthermore, an acetylene inhibition assay was conducted to establish whether the decrease in N_2_O concentrations was due to assimilation or reduction. We herein report the taxonomic affiliation and optimal pH conditions required for N_2_O reduction by this isolated N_2_O-reducing bacterium.

Andisol was collected on April 14, 2016 from a pasture farmland and the maize field at the Hokkaido University Shizunai Experimental Livestock Farm (Shinhidaka, Hokkaido, Japan [42°25'9"N, 142°29'1"E]) (Katayanagi *et al.*, 2008). Soil samples were collected at a depth of 0–10‍ ‍cm and used in the N_2_O reduction assay and the isolation of microorganisms. We used soil from the maize field. The soil suspension was prepared as described previously (Hashidoko *et al.*, 2008).

Winograsky’s mineral solution containing 0.5% (w/v) sucrose and 5‍ ‍mM KNO_3_ (0.52‍ ‍g L^–1^) was used as the medium in the culture-based N_2_O reduction assay (Hara *et al.*, 2009; Nie *et al.*, 2015). Since pH plays a key role in the emission of N_2_O (Nie *et al.*, 2015), the pH of the solution was adjusted to various values (4.5, 5.0, 5.5, 6.0, 6.5, 7.0, 7.5, 8.0, 8.5, and 9.0) using 2 M H_2_SO_4_ and 1 M KOH that was gelled with 0.5% (w/v) gellan gum and then autoclaved. The same medium was used in subsequent experiments. N_2_O levels were measured as described in a previous study (Nie *et al.*, 2015). N_2_O was emitted in the culture at pH 4.5–7.5 (Fig. 1). However, N_2_O emissions decreased in the culture at pH 8.5. This decrease in N_2_O emissions indicated the presence of N_2_O-consuming microorganisms. Therefore, we focused on this culture and isolated the bacterium from N_2_O-consuming microorganisms.

To screen for N_2_O-consuming microorganisms, colonies were isolated as described in a previous study (Nie *et al.*, 2015). Fifteen distinguishable bacterial colonies, marked A to O, were identified. Standard N_2_O gas (GL Sciences) was injected using a gas-tight syringe into the headspace of gas‍ ‍chromatography vials to a final concentration of 2,000‍ ‍ppmv. After incubations for 0, 1, and 4 days at pH 8.5, N_2_O concentrations in the headspace gas were measured. The results obtained showed that strain A (Sac-f1) exhibited the greatest consumption of N_2_O (Fig. 2).

To examine the potential of N_2_O reducers to reduce N_2_O to N_2_, 10% volume (2.25‍ ‍mL) acetylene gas and N_2_O (12,000 ppmv) were injected into the headspace of the assay vials immediately after the inoculation of the isolated bacterium, and media were then incubated at 25°C for 0, 1, 2, 3, and 6‍ ‍weeks. The concentration of N_2_O was measured after these incubation periods. N_2_O concentrations did not decrease with the acetylene gas treatment, which confirmed that the bacterium reduced N_2_O (Fig. 3). Based on the results of OD_660_ measurements in the medium, when N_2_O was injected into gas chromatography vials as the only nitrogen source, this bacterium displayed no growth. This result indicated that this bacterium did not use N_2_O as a nutrient. Furthermore, N_2_O concentrations did not increase during the incubation with the acetylene gas treatment (Fig. 3). Therefore, the bacterium reduced, but did not assimilate, N_2_O and did not denitrify NO_3_^–^ to N_2_O.

The DNA of this bacterium was extracted using an Isoplant II DNA Extraction kit (Nippon Gene), and the *nosZ* gene was subjected to a polymerase chain reaction (PCR) using *nosZ* gene-specific primers (*nosZ*-1111F and *nosZ*-1773R) (Scala and Kerkhof, 1998). The 16S rRNA region was amplified with PCR using the primers 27F and 1525R (Lane, 1991; Weisburg *et al.*, 1991). PCR amplicons using the specific primers were purified by agarose gel electrophoresis. *Pseudomonas denitrificans* NBRC 12442 was used as the positive control. This bacterium did not have a *nosZ* gene (Fig. 4), and the region of 16S rRNA was successfully amplified from the DNA template.

The 16S rRNA sequence of the isolated bacterium was highly homologous to those of the species belonging to *Chitinophagaceae*. The closest species to the isolated bacterium was *Chitinophaga eiseniae* (96.35% similarity). A phylogenetic analysis was performed based on the neighbor-joining method using MEGA X (Kumar *et al.*, 2018). The sequences of the species belonging to *Chitinophagaceae* were retrieved from the GenBank database. A similar phylogenetic analysis was performed using the 16S rRNA sequence data of previously characterized bacteria showing an atypical *nosZ* gene (Liu *et al.*, 2008; Sanford *et al.*, 2012; Jones *et al.*, 2013; Park *et al.*, 2017; Hallin *et al.*, 2018) to identify the taxonomic group of the isolated bacterium. We reviewed these studies for species with an atypical *nosZ* gene in cases where 16S rRNA sequence data were not available. Consequently, this isolated bacterium belonged to the genus *Chitinophaga* (Fig. 5A). However, it was not reported whether the bacteria from this family belonged to clade II *nosZ* (Fig. 5B).

To assess the effects of pH on N_2_O reduction by the isolated bacterium, the pH of the media was adjusted to various values (4.5, 5.0, 5.5, 6.0, 6.5, 7.0, 7.5, 8.0, 8.5, and 9.0), followed by incubations for 0, 1, 2, and 3‍ ‍weeks. N_2_O was injected as described earlier. This isolated bacterium reduced N_2_O at pH in the range of 4.5 to 9.0, with the optimum pH being 8.5 (Fig. 6). Previous studies reported that soil microorganisms belonging to clade II reduce N_2_O at pH 7.0–7.5 (Liu *et al.*, 2008; Sanford *et al.*, 2012; Jones *et al.*, 2013; Park *et al.*, 2017; Hallin *et al.*, 2018), whereas the isolated bacterium in the present study reduced N_2_O under alkaline rather than neutral conditions (Fig. 6).

The present results clearly demonstrated that the isolated bacterium did not assimilate N_2_O, but reduced N_2_O to N_2_. The results of the phylogenetic tree analysis revealed that this bacterium was an unknown species belonging to *Chitinophagaceae* and reduced N_2_O at high pH (8.5). Since the application of nitrogen fertilizers, such as urea, to farmlands results in the largest increase in pH (Black *et al.*, 1985) and accelerates N_2_O emissions (Zhou *et al.*, 2015), a fertilizer inoculated with this isolated bacterium may be used to suppress the N_2_O flux from agricultural soil. Further investigations, draft genome analyses, and measurements of enzyme activity are needed to clarify the genetic background of this isolated bacterium.

## Nucleotide sequence accession number

The 16S rRNA sequence obtained in the present study has been deposited under the following GenBank/ENE/DDBJ accession number: LC554186.

## Citation

Takatsu, Y., Miyamoto, T., and Hashidoko, Y. (2020) An Unknown Non-denitrifier Bacterium Isolated from Soil Actively Reduces Nitrous Oxide under High pH Conditions. *Microbes Environ ***35**: ME20100.

https://doi.org/10.1264/jsme2.ME20100

## Figures and Tables

**Fig. 1.**
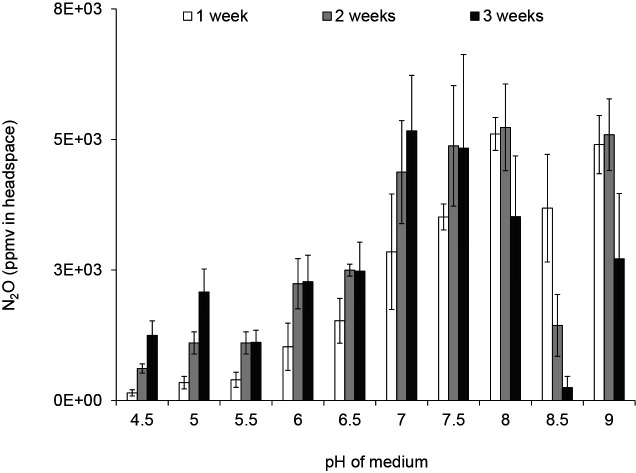
Responses of N_2_O-consuming communities in the soil suspension to optimal pH. The soft gel medium for the culture-based N_2_O reduction assay was adjusted to alternative pH values (4.0, 4.5, 5.0, 5.5, 6.0, 6.5, 7.0, 7.5, 8.0, 8.5, and 9.0), and the soil suspension was incubated at 25°C for 3‍ ‍weeks. Error bars indicate SE (*n*=3).

**Fig. 2.**
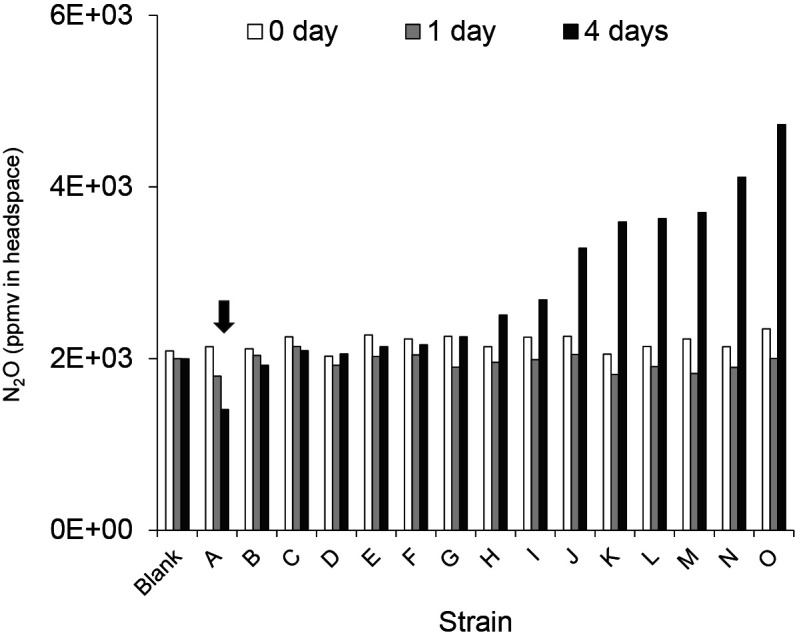
N_2_O-consuming activities of the isolated bacterium in the N_2_O reduction assay. The N_2_O reduction activities of 15 isolates were tested together with a blank in the soft gel medium for the culture-based N_2_O reduction assay (pH 8.5). Among those tested, one isolate (marked as A) reduced the concentration of N_2_O from the background levels of N_2_O (2,000 ppmv) and media were incubated at 25°C for 4 days before N_2_O (approximately 2,000 ppmv) was injected into gas chromatography vials. Blank indicates no bacterial inoculation treatment. The arrow indicates the greatest consumption of N_2_O (A=Sac-f1).

**Fig. 3.**
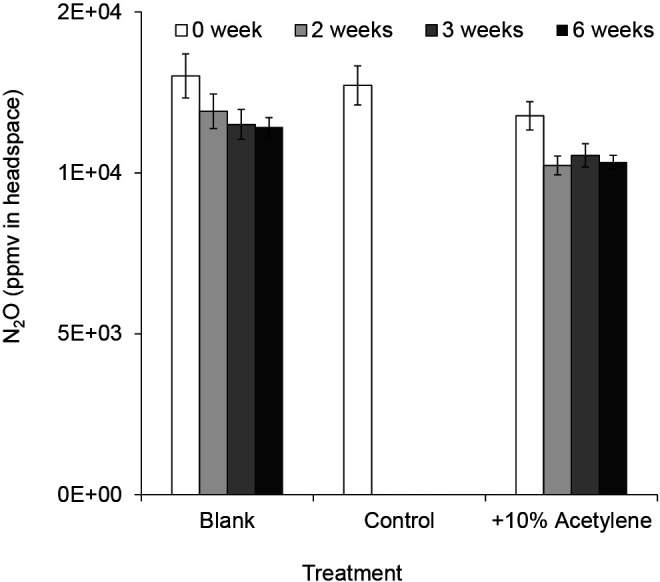
Responses of N_2_O reducers to 10% acetylene gas. The soft gel medium for the culture-based N_2_O reduction assay was adjusted to pH 8.5, and media were incubated at 25°C for 6‍ ‍weeks before N_2_O (about 10,000 ppmv) was injected into gas chromatography vials. Control indicates no acetylene treatment, and +10% acetylene indicates the acetylene treatment. Blank indicates no bacterial inoculation treatment. Error bars indicate SE (*n*=3).

**Fig. 4.**
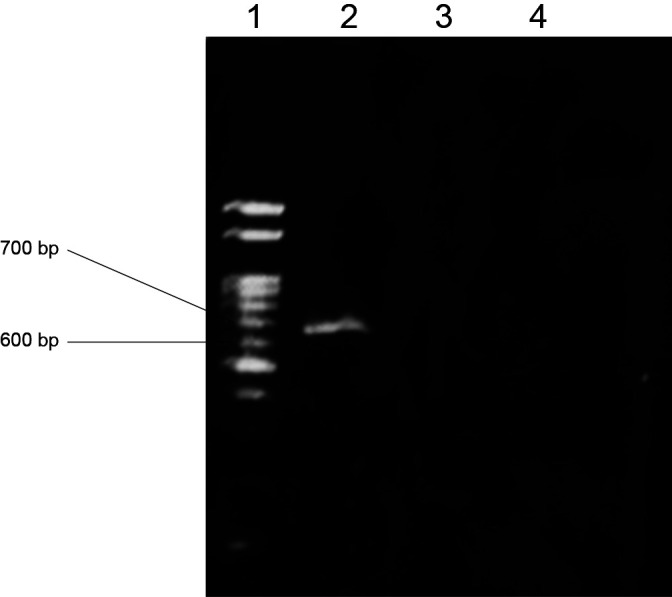
Agarose gel showing the species-specific amplification of the 662-bp fragment. Fluorescence and related species obtained using the primers nosZ 1111 F and nosZ 1773 R. Lane 1: marker gene, lane 2: *Pseudomonas denitrificans* NBRC 12442 (positive control), lane 3: isolated bacterium, and lane 4: Blank.

**Fig. 5.**
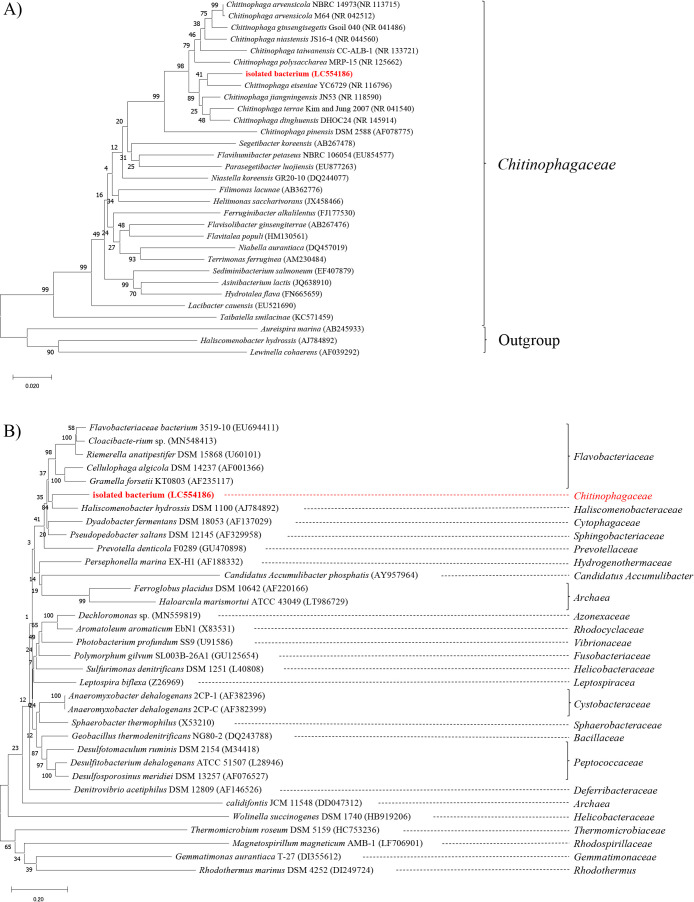
The neighbor-joining tree shows phylogenetic relationships of the isolated bacterium. Similarity and distance matrices were calculated using MEGA X. The phylogenetic tree was constructed based on available 16S rRNA sequences. A) Phylogenetic tree with references from *Chitinophagaceae*. B) Phylogenetic tree with references from the atypical *nosZ* clade. We used the neighbor-joining method with 1,000 bootstrap replicates. The scale bar represents the expected number of changes per sequence position.

**Fig. 6.**
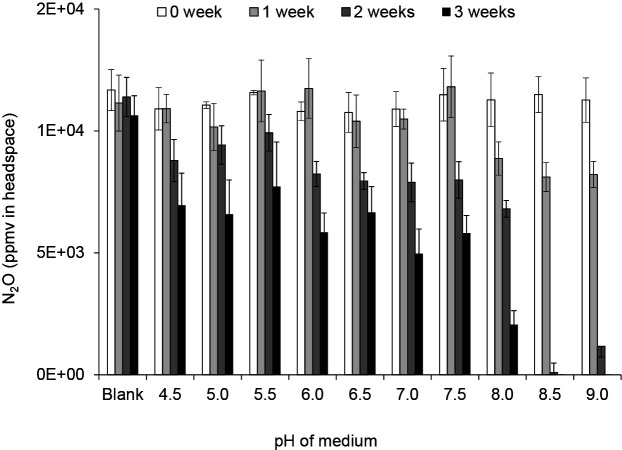
Responses of the isolated bacterium, a N_2_O reducer, to optimal pH. The soft gel medium for the culture-based N_2_O reduction assay was adjusted to alternative pH values (4.0, 4.5, 5.0, 5.5, 6.0, 6.5, 7.0, 7.5, 8.0, 8.5, and 9.0), and media were incubated at 25°C for 3‍ ‍weeks before N_2_O (approximately 10,000 ppmv) was injected into gas chromatography vials. Blank indicates no bacterial inoculation treatment. Error bars indicate SE (*n*=3).
